# Evaluation of digital radiography practice using exposure index tracking

**DOI:** 10.1120/jacmp.v17i6.6082

**Published:** 2016-11-08

**Authors:** Alexander W. Scott, Yifang Zhou, Janet Allahverdian, Jessica L. Nute, Christina Lee

**Affiliations:** ^1^ Department of Radiation Safety Cedars‐Sinai Medical Center Los Angeles CA USA; ^2^ Department of Imaging Cedars‐Sinai Medical Center Los Angeles CA USA; ^3^ Department of Biomedical Sciences Cedars‐Sinai Medical Center Los Angeles CA USA

**Keywords:** digital radiography, radiation dose, quality assurance program, exposure index

## Abstract

Some digital radiography (DR) detectors and software allow for remote download of exam statistics, including image reject status, body part, projection, and exposure index (EI). The ability to have automated data collection from multiple DR units is conducive to a quality control (QC) program monitoring institutional radiographic exposures. We have implemented such a QC program with the goal to identify outliers in machine radiation output and opportunities for improvement in radiation dose levels. We studied the QC records of four digital detectors in greater detail on a monthly basis for one year. Although individual patient entrance skin exposure varied, the radiation dose levels to the detectors were made to be consistent via phototimer recalibration. The exposure data stored on each digital detector were periodically downloaded in a spreadsheet format for analysis. EI median and standard deviation were calculated for each protocol (by body part) and EI histograms were created for torso protocols. When histograms of EI values for different units were compared, we observed differences up to 400 in average EI (representing 60% difference in radiation levels to the detector) between units nominally calibrated to the same EI. We identified distinct components of the EI distributions, which in some cases, had mean EI values 300 apart. Peaks were observed at the current calibrated EI, a previously calibrated EI, and an EI representing computed radiography (CR) techniques. Our findings in this ongoing project have allowed us to make useful interventions, from emphasizing the use of phototimers instead of institutional memory of manual techniques to improvements in our phototimer calibration. We believe that this QC program can be implemented at other sites and can reveal problems with radiation levels in the aggregate that are difficult to identify on a case‐by‐case basis.

PACS number(s): 87.59.bf

## I. INTRODUCTION

In the process of transitioning from screen‐film to computed radiography (CR) and then to digital radiography (DR), “exposure creep”^1^, ^2^, ^3^ has become an issue. This term refers to the fact that digital images, relative to screen‐film, have a wider range of exposure levels producing acceptable contrast and this contrast does not deteriorate with a marginal increase of exposure. Since there was no loss of contrast for progressively increasing exposure levels (which at the same time reduces the image noise), there has been a bias towards doing so. By comparison, screen‐film exhibits a loss of contrast when the exposure lies on the shoulder region of the Hurter‐Driffield (H‐D) curve,^(4)^ so that higher exposure levels result in worse image quality. For those institutions that have goals for optimization of image quality and patient dose (typically given as “as low as reasonably achievable”, or ALARA^(5)^) and use digital radiography, it has been necessary to find a way to determine optimal exposure techniques to prevent exposure creep. Then, having set ALARA targets for some exposure metric at an institution, a QC program can be established to monitor whether its X‐ray imaging program has met those targets.^(6)^ Since the direct link between exposure levels and optimum image quality that was present in screen‐film was broken for digital imaging, the community endorsed the use of an imaging metric to reflect radiation levels.^(7)^ Manufacturers of CR plates introduced a variety of exposure metrics, some of which were determined by properties other than radiation exposure, such as postprocessing of the image.^(8)^ The American Association of Physics in Medicine (AAPM) and International Electrotechnical Commission (IEC) recommended the use of a standard exposure index that is dependent on the air‐kerma incident at the detector for a defined beam quality, and recommended its use in DR as well.^1^, ^9^ One example of such a metric is Carestream's (Carestream Health, Rochester, NY) exposure index (EI), which has been defined as:

$$EI=1000*{\text{log}}_{10}(\frac{X}{X_{0}})+2000$$

where *X* is the incident exposure in units of mR and $X_{0}$ is defined as 1.0 mR (8.7μGy)^(10)^ This relationship between EI and exposure is defined specifically for an X‐ray beam at 80 kVp and with an additional filtration of 0.1 mm Cu and 1.0 mm Al.^(1)^ Although the IEC definition of exposure index is likely to be used across platforms in the future, the Carestream EI was used during this project and will be the EI definition referenced henceforth.

Although the dependence of EI on exposure to the detector is clear in principle, in practice it may not be obvious how the software determines the signals from which to calculate EI. A histogram is made of signal strengths in the preprocessed image and the average value is used to compute the EI. The histogram contents may come from the entire image, or from a user‐defined ROI, or a region derived from organ segmentation. In clinical practice, it is organ segmentation that determines EI and choosing a different organ or anatomy of interest will change the displayed EI;^(11)^ therefore, reproducibility will depend on consistent anatomy segmentation and patient positioning.

A viable approach to achieving consistent image quality over a wide range of patient thicknesses is to use the exposure index as a guide to maintaining a consistent air‐kerma incident to the receptor.^(12)^ The exposure index value corresponding to that incident air kerma is the target exposure index, which can be realized either by using a calibrated automatic exposure control (AEC) system or proper manual technique. The AEC of an X‐ray unit depends on phototimers to terminate the X‐ray exposure, and can be tuned so that the detector exposure on termination meets the institutional target.^(13)^ However, the exposure termination is based on anatomy directly over the phototimer, so if organ segmentation includes nonhomogeneous body thicknesses then the EI will have a range of values based purely on patient positioning.

As an alternative to relying on AEC, the technologist can choose to use a manual technique, either according to a posted chart or according to a technologist's best assessment of the appropriate technique based on body habitus and other factors. Accurate manual technique requires knowledge of the appropriate technology as screen‐film, CR, and DR require different techniques to achieve optimum image quality. Thus, there will be a range of EI values for properly exposed images (due to averaging over a segmented region) and there may be values representing inappropriate manual techniques, poor positioning of anatomy, or a suboptimal AEC calibration.

It may be difficult to identify the reason for an individual image to have an EI value outside the range set by the institution — whether it was due to normal variations in a correctly exposed image, a one‐time error in exposure, or a systematic deviation from best practice where an intervention may be useful. However, it is possible to use collected exposure index statistics to analyze exposure distributions and differentiate trends by technologist, X‐ray unit, or protocol. The ability to identify outliers in practice allows for targeted interventions. There are examples of QC programs using automated collection methods to identify trends in computed tomography (CT),^14^, ^15^ and in CR tracking rejects and exposure levels over time.^16^, ^17^, ^18^, ^19^ AAPM Task Group 151^(6)^ discusses the appropriate quality assurance elements in a digital radiography program, including analysis of exposure levels. AAPM Task Group 151 suggests a variety of analyses of collected exposure data, including looking at variations in performance between units, technologists, and protocols.

This paper describes an implemented QC program for the clinical medical physicist in digital radiography analyzing the distributions of exposure data to better understand the causes of variation in detector exposures within a specific institution. This QC program included setting institutional guidelines for exposure levels, remotely downloading statistics for analysis, and identifying sources of high patient exposures. This paper will include examples of successful interventions where the median patient dose for the reviewed protocols was reduced by 40%, as evidenced by the drop in median EI. Other clinical medical physicists may desire to implement a similar QC program for digital radiography at their institutions.

## II. METHODS

Our QC program for digital radiography involved calibration of the detector exposure metric, calibration of the AEC phototimer, and remote collection of exposure statistics from the digital detectors. The collected data were then processed to provide information on trends in exposure levels by body protocol and technologist and the trends were compared between units. After interventions were made, the data were monitored to verify the desired change.

In this study, QC records from both Carestream DR retrofit units and Carestream integrated X‐ray units were used. The Carestream DR retrofit kits were combined with GE Advantx (General Electric, Fairfield, CT) X‐ray units. This retrofit was used to convert existing CR X‐ray rooms to DR by using a cassette‐sized digital detector that could be inserted into the bucky. Image acquisition was triggered by the generator, but the generator information was otherwise separate and no information on kVp or mAs was stored in the DR image DICOM header metadata. Likewise, no information on use of manual versus AEC mode was preserved. The GE Advantx console has programmable manual and AEC techniques. The Carestream integrated units used were DRX Evolution, which have an integrated detector and generator. These consoles also have programmed manual and AEC techniques, and the generator information was stored in the DICOM header. Both retrofits and integrated units use Carestream DRX 1‐C digital detectors, which utilize a cesium iodide crystal as a detection medium.

### A. Acceptance testing of digital detectors

#### A.1 Exposure index calibration

As part of the clinical job of acceptance testing the DRX detectors, it was necessary to evaluate the EI accuracy for the new detectors and to calibrate the phototimers. The EI calibration accuracy was verified according to the procedure and geometry in Samei et al.^(8)^ Following the manufacturer's specifications, the detectors were exposed with the following X‐ray beam: 80 kVp with filtration of 1mmAl+0.5mm Cu. A correction factor was determined to relate the measured exposures at the center of the beam and at the periphery (so that the chamber did not affect the EI of later exposures). The time‐integrated tube current was varied between 1 and 6.4 mAs and the resultant exposure was measured at the periphery of the beam using a Radcal Accu‐Pro ion chamber (Radcal Corp., Monrovia, CA). The exposure at the plate was determined using appropriate geometric corrections and the EI was calculated using Eq. (1).

The displayed EI value for each exposure was found by entering a “superuser” mode in the software and placing a predefined ROI at the center of the image, using a “Pattern” view. This EI value was specific to the uniform region covered by the ROI and did not involve any segmentation, which performs poorly when exposing a nonanatomical phantom. In the absence of manufacturer specifications for agreement between displayed and calculated EI values, a maximum of 10% deviation was allowed and all measurements passed this requirement before the detector entered clinical use.

#### A.2 Phototimer calibration

After verifying the EI calibration, the phototimers of each unit were calibrated with the assistance of the field service engineers according to manufacturer instructions. This process involved exposing incremental thicknesses of Lucite (between 5 cm and 20 cm) using AEC mode and the center phototimer, according to prescribed kVp settings between 50 kVp and 130 kVp. The images were created using a “Pattern” view and the EI was measured using an ROI at the center of the image. The phototimer performance was adjusted so as to meet the institution's target EI of 1600 at each kVp within an EI range of ±150. Carestream's instructions specified that AEC performance should ideally meet the EI target within ±20, corresponding to 5% variation in exposure. However, we observed at our institution that the AEC performance across all kVp values could not meet this strict requirement and that an EI range of ±150 was realistic and acceptable.

The first Carestream DRX 1‐C detectors at our institution were part of retrofit kits. The target EI for the retrofit detectors was initially set for 1600, which was conservatively chosen in relation to the EI target for Carestream CR. Later, when the Carestream Evolution X‐ray units were installed, the integrated unit phototimers were calibrated to a nominal incident air kerma of 2.5μGy, corresponding to a target EI of 1450. This value was consistent with the incident air kerma recommended in private communication by Siemens (Siemens Medical Solutions, Forchheim, Germany) for their Drixell DR detector. After the reading physicians reported to the QC team that the image quality at target EI=1400 was acceptable on this unit, a consistent EI target of 1400 was implemented in November 2014 for all X‐ray units, patients, body parts, and views. The phototimers were recalibrated to an EI of 1400 following the previously described procedure. Subsequent results have indicated that anatomy‐ or task‐specific EI targets are appropriate, but the development of such targets was outside the scope of this project.

### B. QC data collection

The detectors were configured during acceptance testing (ranging from 2011 through 2014 as new detectors were acquired) for remote downloading of exposure statistics collected over time. This allowed a specified user to log in to the host terminal for the digital detector and run DirectView (Carestream Health, Inc.), which is a software interface for management and download of data. The user could then download image acquisition statistics (but not patient images, or modify service settings). The data were provided in a spreadsheet format for analysis in Microsoft Excel (Microsoft, Redmond, WA). Data fields for each exposure event included date and time, body part and projection, “Tech ID,” “Exposure Index,” and reject information; the technical parameters “kVp” and “mAs” were not available from this software. For the QC process, records were extracted on a monthly basis using the remote‐access software and reviewed for trends and incidents involving image reject rates and median exposure index values for protocols. Feedback was then sent to the institution on retake rates and median EI values per protocol over time.

### C. Clinical exposure data analysis

Of special interest was the performance of the retrofit X‐ray units before and after recalibration to an EI of 1400 and the relative performance compared to the integrated X‐ray units. An IRB exemption was granted for this research, since no patient information was used and the data were drawn retrospectively from records stored on the DRX detectors. The EI results were drawn from abdomen and chest protocol records; these two protocols were of interest because of higher radiation levels (relative to extremities or head) to radiosensitive organs and the large number (>100) of studies per month per unit to analyze. The data came from two GE Advantx retrofit units, which were recalibrated to an EI of 1400 in November 2014, and two Carestream integrated X‐ray units, which were calibrated at installation to an EI of 1400. These four units were well suited to comparison since they were located in two distinct radiology departments at the same site, labeled as departments “A” and “B,” with one integrated and one retrofit unit each. The technologists of departments A and B were separate and did not interchange, so they have been treated as independent. Table 1 shows the relationship of the units A1, A2, B1, and B2; “A” or “B” for the department and “1” or “2” for integrated or retrofit, respectively.

Two data series were studied for each protocol: a 2014 dataset covering January through November 2014 and a 2015 dataset covering December 2014 through May 2015. All views for each protocol were included in the results, which were binned and plotted as histograms. All histograms were normalized to the number of images in the sample, so that the area under each curve was unity. Histograms and statistical quantities were calculated using MS Excel. Comparisons were made between the two integrated units (A1 and B1), between the two retrofit units (A2 and B2), and between unit types within each department (A1 and A2, B1 and B2), to evaluate how the clinical exposure levels changed between the 2014 and 2015 datasets. Finally, we planned to make an intervention if the results indicated that the phototimer recalibration was ineffective in producing consistent mean EI between units; in this case, the change was considered effective if the new mean EI was within ±150 of the institution's target EI (i.e., between 1250 and 1550), where ±150 was the accuracy of the phototimer recalibration. Protocols were considered on‐target if the mean EI each month was within ±300, where twice the variability was allowed due to the smaller sample size.

**Table 1:** Comparison among four units: Unit A1 is the Carestream integrated unit in department A, unit A2 is the GE retrofit unit in department A, unit B1 is the Carestream integrated unit in department B, and unit B2 is the GE retrofit unit in department B. The symmetry allows for performance comparison between different work areas for the same model and type of X‐ray unit (e.g., unit A1 vs. unit B1), or between models within the same work area (e.g., unit A1 vs. unit A2)

	*Dept. A*	*Dept. B*
Carestream Evolution (integrated)	Unit A1	Unit B1
GE Advantix (retrofit)	Unit A2	Unit B2

## III. RESULTS

### A. Acceptance testing results

#### A.1 Exposure index accuracy

The acceptance testing results for assuring exposure index accuracy for unit A1 are given in Table 2. The accuracy of the displayed EI compared to measured exposure was within 10% for all exposure levels, with the greatest deviation at the lowest mAs (8.20%) and the least deviation at higher mAs values (2.80%). All other Carestream DRX 1‐C detectors met the same acceptance requirements and their results are omitted for brevity.

**Table 2:** Results of EI accuracy testing during acceptance of detector for unit A1 (department A integrated). The geometry used was 170 cm SID, 112 cm SCD, and the ratio of measured exposure at the center of the beam and at the periphery was 1.112. The beam used was the Kodak reference beam, which was 80 kVp filtered by 0.5 mm Cu and 1.0 mm Al. The exposure at the detector was calculated using the appropriate geometric correction and the ratio of center to periphery exposure. The displayed EI was found using the ROI method and the calculated EI was found using Eq. (1)

*mAs*	*X at Chamber (mR)*	*X at Detector (mR)*	*Displayed EI*	*Calculated EI*	*Deviation*	*% Error*
1.0	0.35	0.169	1328	1228	100	8.2%
1.6	0.64	0.309	1572	1490	82	5.5%
2.0	0.89	0.429	1703	1633	70	4.3%
2.5	1.20	0.579	1824	1763	61	3.5%
3.2	1.59	0.767	1943	1885	58	3.1%
4.0	2.04	0.984	2054	1993	61	3.1%
5.0	2.70	1.303	2173	2115	58	2.8%
6.4	3.46	1.669	2284	2223	61	2.8%

#### A.2 Phototimer calibration results

The phototimer calibration results for unit A1 at acceptance are given in Table 3. The target EI was 1400 with an acceptable range of ±150. The phototimer calibration results for unit A2, showing the resultant EI values at initial recalibration (prior to Nov. 2014) and at final recalibration (Nov. 2014), are given in Table 4. All other phototimers met the same calibration requirements and their results are omitted for brevity.

**Table 3:** Results of table phototimer calibration (target EI=1400) during acceptance testing of unit A1 (department A integrated). The beam used was the clinical beam with no additional filtration and exposures were made in AEC mode. The central phototimer was used for AEC. The displayed EI was found using the ROI method

*Phantom Height (cm)*	*kVp*	*mAs*	*Displayed EI*	*Deviation From Target*	*% Error*
5	50	4.2	1393	‐7	‐0.5%
7.5	55	4.5	1398	‐2	‐0.1%
7.5	65	2.2	1388	‐12	‐0.9%
12.5	75	3.0	1401	1	0.1%
12.5	85	1.7	1411	11	0.8%
12.5	95	1.2	1437	37	2.6%
20	110	2.5	1479	79	5.6%
20	130	1.5	1496	96	6.9%

**Table 4:** Results of table phototimer calibration during initial phototimer recalibration (target EI=1600) and final recalibration (target EI=1400) of unit A2 (department A retrofit). The beam used was the clinical beam with no additional filtration and exposures were made in AEC mode. The central phototimer was used for AEC. The displayed EI was found using the ROI method

		*Initial Calibration*		*Final Calibration*	
*Phantom Height (cm)*	*kVp*	*Displayed EI (in.)*	*Target Dev. (in.)*	*Displayed EI (fin.)*	*Target Dev (fin.)*
5	50	1579	‐21	1427	27
7.5	55	1562	‐38	1401	1
7.5	65	1537	‐63	1401	1
12.5	75	1540	‐60	1360	‐40
12.5	85	1518	‐82	1365	‐35
12.5	95	1491	‐109	1357	‐43
20	110	1532	‐68	1310	‐90
20	130	1551	‐49	1337	‐63

#### B. Clinical exposure data analysis results

**Figure 1:**
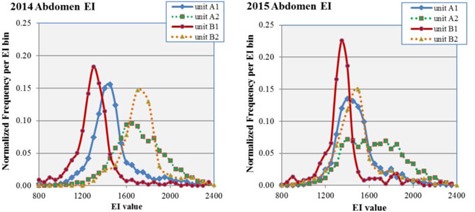
EI distributions for abdomen studies for units A1, A2, B1, and B2 for datasets 2014 (left) and 2015 (right). Units A1 and B1 are the integrated units while A2 and B2 are the retrofit units, from departments A and B, respectively. Distributions are normalized to the number of exams so that the area under each curve is unity. The 2014 dataset is prior to the phototimer recalibration and the 2015 dataset is after.

**Figure 2:**
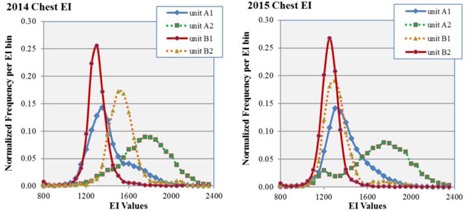
EI distributions for chest studies for units A1, A2, B1, and B2 for datasets 2014 (left) and 2015 (right). Units A1 and B1 are the integrated units while A2 and B2 are the retrofit units from departments A and B, respectively. Distributions are normalized to the number of exams so that the area under each curve is unity. The 2014 dataset is prior to the phototimer recalibration and the 2015 dataset is after.

The statistics for abdomen protocols based on the 2014 and 2015 datasets are presented in Table 5 and include the number of images, the mean, median, and standard deviation of the EI for each dataset, the skewness, and the excess kurtosis. The equivalent information for chest protocols based on the 2014 and 2015 datasets is presented in Table 6. Comparisons of the EI distributions across the four units A1, A2, B1, and B2 are presented for abdomen and chest protocols in Figs. 1 and 2, respectively.

In the 2014 abdomen dataset, the mean EI values of the retrofit units A2 and B2 are similar (1693 versus 1678) although the excess kurtosis is very different (0.38 versus. 4.92), resulting in a much wider distribution for B2 compared to A2. By comparison to the two reftrofits, the two integrated units, A1 and B1, have lower mean EI (1451 and 1300, respectively) and also excess kurtosis >3.0. In the 2015 abdomen dataset, the mean EI values of the two integrated units A1 and B1 (1427 and 1369, respectively) moved closer to the target EI of 1400. The recalibration of the B2 phototimer resulted in a lower mean EI of 1457, indicating much better agreement between the two types of units. The mean EI of A2 remained above an EI of 1600 at 1625. The EI distribution for A2 is so broad (excess kurtosis of ‐0.32) that it might be better fit by two overlapping gaussians.

**Table 5:** Comparison among four units: unit A1 (department A integrated), unit A2 (department A retrofit), unit B1 (department B integrated), and unit B2 (department B retrofit) for 2014 and 2015 datasets, abdomen protocols. Displayed values are the number of images, the mean and median EI values, and the standard deviation (SD) of the EI values. The skewness and excess kurtosis of the distribution are also given

	*Unit A1*	*Unit A2*	*Unit B1*	*Unit B2*
*2014 Abdomen EI*				
# images	2,262	4,020	576	313
EI mean	1451	1693	1300	1678
EI median	1417	1677	1285	1672
EI SD	229	235	236	198
Skewness	+1.15	+0.11	+2.35	+0.15
Kurtosis	+3.52	+0.38	+10.78	+4.92
*2014 Abdomen EI*				
# images	1,822	2,800	381	241
EI mean	1427	1625	1369	1457
EI median	1431	1614	1328	1417
EI SD	200	261	256	248
Skewness	+1.17	+0.22	+2.20	+1.42
Kurtosis	+2.79	‐0.32	+7.12	+3.42

**Table 6:** Comparison among four units: unit A1 (department A integrated), unit A2 (department A retrofit), unit B1 (department B integrated), and unit B2 (department B retrofit) for 2014 and 2015 datasets, chest protocols. Displayed values are the number of images, the mean and median EI values, and the standard deviation (SD) of the EI values. The skewness and excess kurtosis of the distribution are also given

	*Unit A1*	*Unit A2*	*Unit B1*	*Unit B2*
*2014 Chest EI*				
# images	5,187	7,151	4,589	2,006
EI mean	1441	1772	1264	1563
EI median	1398	1787	1255	1556
EI SD	205	238	124	168
Skewness	+0.84	‐0.44	+1.83	+0.05
Kurtosis	+1.47	+0.70	+21.67	+4.84
*2015 Chest EI*				
# images	5,629	5,810	2,874	1,816
EI mean	1427	1730	1279	1353
EI median	1397	1758	1276	1331
EI SD	186	274	115	186
Skewness	+0.59	‐0.45	‐0.21	+1.22
Kurtosis	+1.46	+0.14	+16.94	+8.80

In the 2014 chest dataset, the EI distributions of the integrated units A1 and B1 both peak between 1300 and 1400 EI, but the difference in skewness (0.84 versus 1.83) and excess kurtosis (1.47 versus 21.67) result in mean EI values that are almost 200 apart. The data for retrofit unit A2 has a negative skewness, low kurtosis, and high mean EI (1772) resulting in a distribution that looks unlike the integrated units. Unit B2 has a similar appearance to B1 and a mean EI (1563) that is between A1 and A2. In the 2015 chest dataset, the EI distribution of retrofit B2 after recalibration has shifted so that the peaks of A1, B1, and B2 lie between EI of 1250 and EI of 1350. Unit A2 is characterized by negative skewness and low kurtosis because there is a secondary peak on the low‐side tail of the main EI distribution; there is very little change between 2014 and 2015 in the primary peak (1800 for both) and mean EI (1772 vs. 1730).

**Figure 3:**
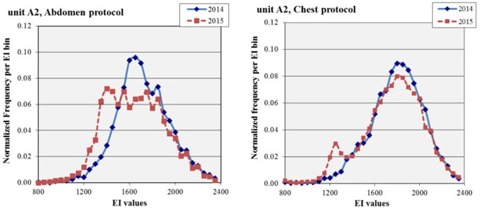
Before‐and‐after EI comparisons for one X‐ray unit. Distribution of EI values for abdomen protocol exams (left) and chest protocols (right) for unit A2 (department A, retrofit Carestream). Both 2014 and 2015 distributions are normalized to the number of exams so that the area under each curve is unity. The phototimer recalibration between datasets 2014 and 2015 has been ineffective in shifting the EI distribution from an EI target of 1600 to a target EI of 1400 in both cases.

Figure 3 shows an overlay of 2014 and 2015 EI data for retrofit unit A2 for abdomen and chest protocols, respectively, for better comparison of the EI distributions before and after recalibration. The histogram of the abdomen protocol EI values for retrofit unit A2 shown in Fig. 3 has notable features. The 2014 data have a small peak on the right shoulder, between EI of 1800 and 1850, representing 49 events or 1.2% of the data. The 2015 abdomen data have a similar distribution to the 2014 data, except that there are fewer events in the bins surrounding the mean value and there is an increase in events on the low shoulder between EI values 1350 and 1500. The median EI values for the 2014 and 2015 datasets are within 70 EI points of each other. A histogram of the chest protocol EI values for retrofit unit A2 is also shown in Fig. 3. The 2014 data have a negative skewness representing the low‐side shoulder. The 2015 data have similar skewness and kurtosis, except for a peak in the bin representing EI values from 1150 to 1300, which was fitted to contain 7.8% of the studies (447 events).

## IV. DISCUSSION

The development and implementation of this QC program has led to important findings. First, the EI distributions of the integrated units A1 and B1 were found to be consistent within 50 EI points between the 2014 and 2015 datasets. Second, the phototimer recalibration was found to produce an effective (as defined in the Methods section) change in the department B retrofit EI values. Finally, the phototimer recalibration was found to not have produced an effective change in the department A retrofit (A2) EI values. In addition to determining the effectiveness of the recalibration, features of the EI distributions provided evidence for how the types of exposures and techniques were employed on these units. The causes for the differing values and features of the EI distributions were further examined in order to make useful interventions.

The EI values for the integrated X‐ray units were straightforward to explain. The integrated units were new installations in 2013, initially calibrated to EI 1400, and given manual technique charts reflecting estimates of kVp and mAs to achieve this EI. Our EI tracking program for the integrated units has shown that the median EI values for tracked protocols were within the target range of 1400±300 on a monthly basis since the beginning of this QC project. Table 7 presents statistics from unit A1 protocols representing 50% of exams; no protocol has a median EI outside the institutional EI range. In 2014 and 2015, between 80% and 85% of all accepted images from the two Carestream integrated units had an EI within the target range. These findings are supportive of the consistency by which the integrated units A1 and B1 have hit the EI target.

**Table 7:** Statistics of sample protocols from 2015 for unit A1. The four protocols listed represent more than 50% of images in that dataset. There were no protocols with median EI outside the institutional range of 1400±300

*Protocol*	*Median EI*	*EI SD*	*Percentage of Dataset*
Chest	1397	186	28.1%
Abdomen	1431	200	9.1%
Knee	1525	162	8.5%
Wrist	1570	170	5.6%

**Figure 4:**
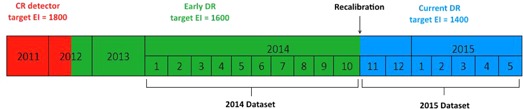
Timeline of EI targets for retrofit units. The 2014 dataset comes from the target EI=1600, while the 2015 dataset comes from target EI=1400.

The two retrofit units presented in this analysis had a very different history from the integrated units. Both retrofits were originally CR, with an original target EI of 1800, then retrofitted with Carestream DR kits and assigned an EI target of 1600. After further testing, the EI target was lowered to 1400. A simple timeline of EI targets over time is shown in Fig. 4. These units therefore have an institutional history of using techniques producing higher radiation levels at the detector, and it is plausible that there is an institutional memory of old manual technique among the technologists that is not relevant to the current technology.

The data for the department A retrofit unit (A2) show evidence indicative of both AEC and manual technique at three different EI points (1800, 1600, and 1400). The use of manual technique is particularly a problem when technology changes; although technologists can and do make good choices of manual technique, these units experienced enough technology and software changes that previously appropriate techniques delivered substantially more radiation than was necessary for diagnostic imaging at the time of this study. For this unit, the 2014 abdomen data peaked between 1600 and 1650 as expected. The small peak at EI 1800 is consistent with the target EI of the unit when it used CR plates and is consistent with the attestation of the technologists that they prefer the manual technique that they learned in the past. The 2015 data were collected after recalibration to the lower EI target and a reminder to the technologists of the institutional policy on using automatic exposure control. The 2015 data appear to be unshifted relative to the 2014 data, although there is evidence of a peak at the new target EI of 1400. For comparison, the department B retrofit abdomen data show a definite shift from previous performance at a target EI of 1600 to the current more acceptable target EI of 1400. Both retrofit units have the same manufacturer, utilize the same type of detector, and were recalibrated on the same day, but the techniques used on each are clearly different.

The results from the chest protocol data showed the same pattern. The 2014 data for the department A retrofit unit (A2) had an EI distribution peaked at 1800, which was higher than the target EI at that time but was consistent with the old CR target EI. After the recalibration, the distribution remained almost the same but with a secondary peak at 1250, consistent with the chest phototimer in A1. The chest protocol in all units produced average EI values below the target, so this peak was consistent with using the calibrated AEC. Again, for comparison, the department B retrofit (B2) chest data showed a definite shift from previous performance at a target EI of 1600 to the current performance at target EI of 1400.

Finally, the fraction of exposures pre‐ and postrecalibration meeting the target EI range of 1400±300 was 38% vs. 41% for the department A retrofit and 53% vs. 68% for the department B retrofit. The average EI for unit A2 did not move much towards the target in the six months following the recalibration. Table 8 presents statistics on unit A2 sample protocols representing 50% of exams; three of those five protocols had a median EI outside the institutional range in the 2015 dataset. Based on the discussion above, it was concluded that the phototimer recalibration of unit A2 had not been effective.

**Figure 5:**
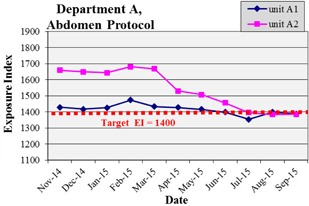
Evidence of lowered radiation dose after intervention. Average EI value per month for abdomen protocol exams for units A1 and A2 (integrated and retrofit units, respectively). The QC team worked with a lead technologist in April to start tracking of EI values by technologists, and an in‐service was delivered in June for lowering the average EI. The EI control is 1400 and is drawn in a dashed line.

After the conclusion of data analysis, the QC team prepared an intervention targeted for department A to achieve the dose savings of department B. A presentation was made comparing performance between X‐ray units in both departments, and departmental support was offered to make changes in practice. An in‐service lecture on the EI metric and dose control was delivered to the technologists, all of whom expressed interest in patient dose reduction. This issue was used as a performance quality improvement (PQI) project and a technologist was chosen to work with the QC team to prepare monthly tracking reports of EI by protocol and unit. Figure 5 is an example of EI tracking by protocol and shows that the department A retrofit median abdomen EI dropped from 1668 in March to 1530 in April and 1456 in June, which corresponds to almost a 40% reduction in exposure. The reduction in April is consistent with the beginning of technologist tracking EI values by hand for a related QC project, and the June reduction is consistent with the in‐service provided. By August, the EI values for the integrated unit and the retrofit unit had converged.

**Table 8:** Statistics of sample protocols from 2015 for unit A2. The five protocols listed represent more than 50% of images in that dataset

*Protocol*	*Median EI*	*EI SD*	*Percentage of Dataset*
Chest	1758	274	23.0%
Abdomen	1614	261	11.1%
Knee	1758	186	8.3%
L‐Spine	1652	257	6.7%
Hip	1770	250	3.9%

## V. CONCLUSION

By reconstructing EI distributions, the QC program identified units meeting a dose target and one unit not meeting the dose target after phototimer recalibration. Based on interviews with technologists and the QC data, the evidence suggests that the technologists in department A were using manual technique with an institutional memory of previous exposure targets. Targeted intervention has lowered department A radiation levels to the institutional target within four months. Each drop of 200 in EI is approximately a 40% reduction in exposure, so instituting a change in practice has had a substantial positive impact on patient radiation dose.

The QC group's findings in this ongoing project have allowed useful interventions to be made: from emphasizing the use of phototimers instead of manual techniques when appropriate, to identifying previously unrecognized problems with phototimer calibration, to raising awareness of exposure metrics and the relation to patient dose. Narrowing the focus to a single group of outliers has allowed for more targeted intervention and education. Monthly tracking of median EI per protocol has documented a change in practice since the interventions, and new goals are being set so that all X‐ray units provide consistent exposures over time. We believe that this QC program should be straightforward to implement for other clinical medical physicists at institutions with Carestream DRX detectors and can reveal problems with radiation levels in the aggregate that are difficult to identify on a case‐by‐case basis.

As further work, the QC group is developing new manual technique charts for the retrofit units that will be based on correlating the EI and AEC technique values recorded in the DICOM image header. Techniques will be collected for large numbers of studies and investigated for statistical groupings. The statistically average techniques for small, medium, and large body habitus will be used to create manual technique charts based on these results, in accordance with the recommendations in the ACR‐AAPM‐SIIM practice guidelines for digital radiography.^(20)^ In addition to the continuing work based on monthly EI tracking and supporting QA development in the institution's digital radiography practice, our group is also working to develop size‐specific EI targets based on constant CNR across patient sizes.

## ACKNOWLEDGMENTS

We would like to acknowledge Donna Earley, Lynne Roy, and Jonathan Wilson for support of our research and to acknowledge our X‐ray imaging division, including Lionel Bravo and Kevin Ching, for their assistance in collecting the data.

## COPYRIGHT

This work is licensed under a Creative Commons Attribution 3.0 Unported License.

## Supporting information

Supplementary Material

Supplementary Material

Supplementary Material

Supplementary Material

Supplementary Material

Supplementary Material

Supplementary Material

Supplementary Material

Supplementary Material
