# Supplementary figures and images for: Evaluation of digital radiography practice using exposure index tracking
Source: J Appl Clin Med Phys. 2016 Nov 8;17(6):343–55. doi: 10.1120/jacmp.v17i6.6082 (PMC5690495; doi:10.1120/jacmp.v17i6.6082)

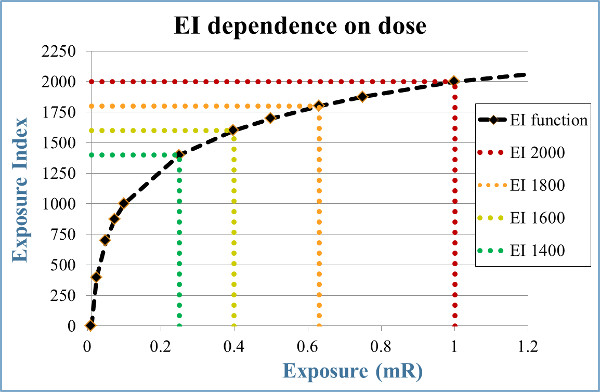

Supplement: Supplementary file 1 — Supplementary Material [file ACM2-17-343-s001.jpg]

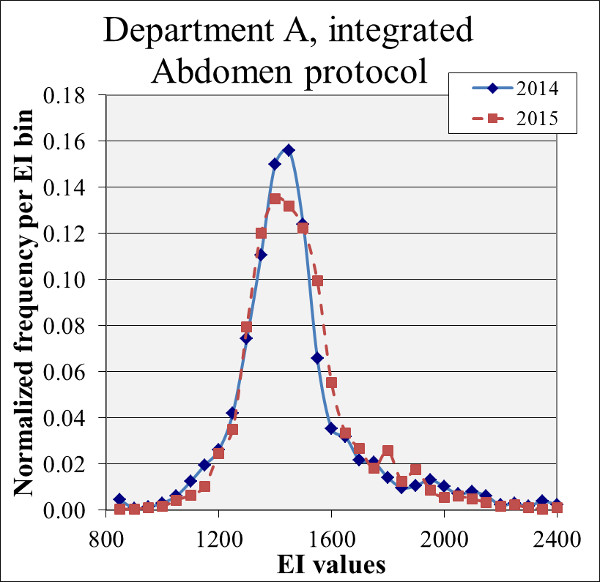

Supplement: Supplementary file 2 — Supplementary Material [file ACM2-17-343-s002.jpg]

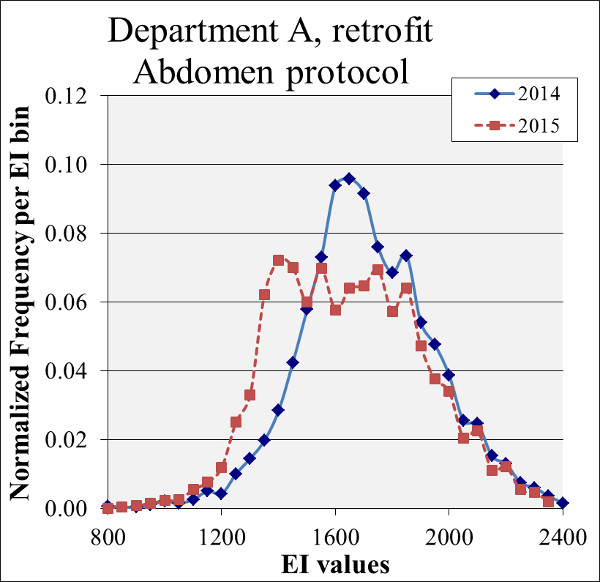

Supplement: Supplementary file 3 — Supplementary Material [file ACM2-17-343-s003.jpg]

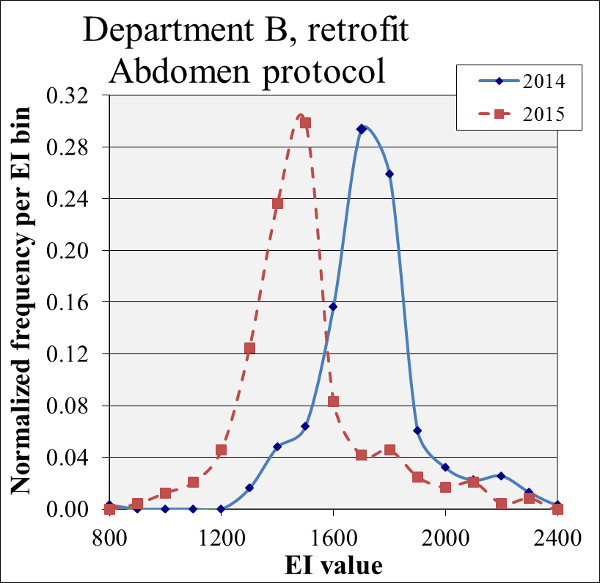

Supplement: Supplementary file 4 — Supplementary Material [file ACM2-17-343-s004.jpg]

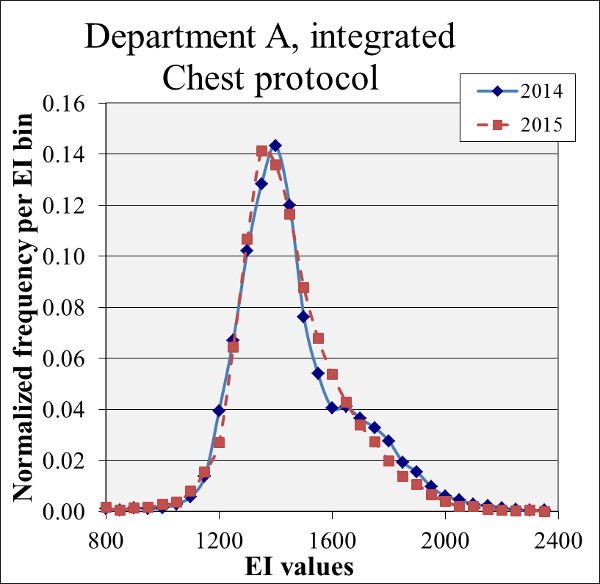

Supplement: Supplementary file 5 — Supplementary Material [file ACM2-17-343-s005.jpg]

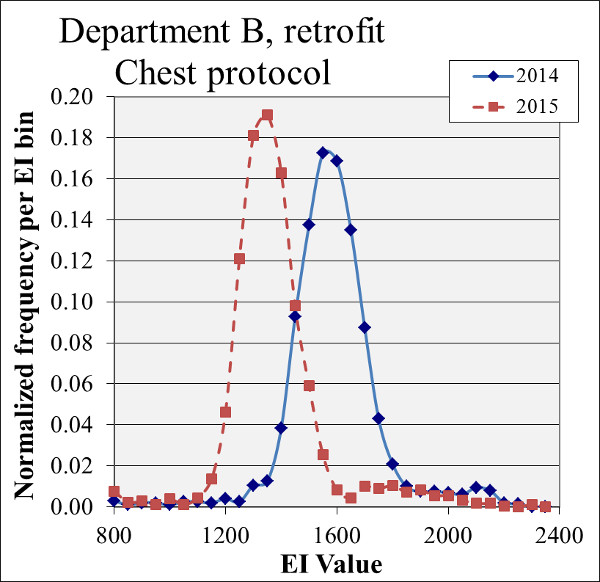

Supplement: Supplementary file 6 — Supplementary Material [file ACM2-17-343-s006.jpg]

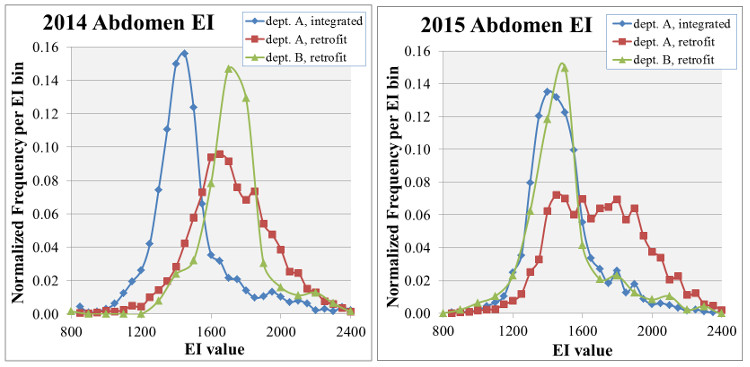

Supplement: Supplementary file 7 — Supplementary Material [file ACM2-17-343-s007.jpg]

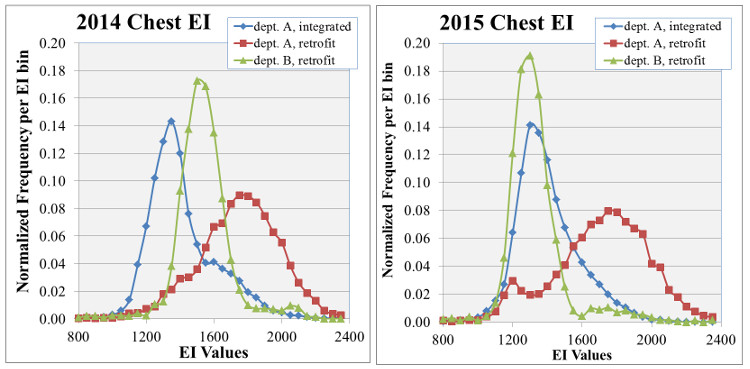

Supplement: Supplementary file 8 — Supplementary Material [file ACM2-17-343-s008.jpg]

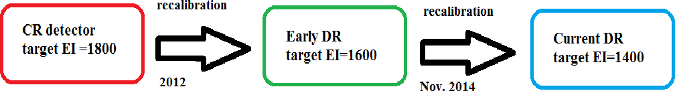

Supplement: Supplementary file 9 — Supplementary Material [file ACM2-17-343-s009.jpg]
